# Parameterization of the vertical distribution of leaf area index (LAI) in rice (*Oryza sativa* L.) using a plant canopy analyzer

**DOI:** 10.1038/s41598-018-24369-0

**Published:** 2018-04-23

**Authors:** Yoshihiro Hirooka, Koki Homma, Tatsuhiko Shiraiwa

**Affiliations:** 1 Graduate School of Agricultural Sciences, Kindai University, Nara, 631-8505 Japan; 2 Graduate School of Agricultural Science, Tohoku University, Sendai, 981-8555 Japan; 3 Graduate School of Agriculture, Kyoto University, Kyoto, 606-8502 Japan

## Abstract

Monitoring the vertical distribution of leaf area index (LAI) is an effective method for evaluating canopy photosynthesis and biomass productivity. In this study, we proposed a novel method to characterize LAI vertical distribution non-destructively by utilizing LAI-2200 plant canopy analyzer, followed by the application of statistical moment equations. Field experiments were conducted with 5 rice cultivars under 2 fertilizer treatments in 2013 and with 3 rice cultivars under 3 plant density treatments in 2014. LAI readings obtained by a plant canopy analyzer for non-destructive stratified measurements were relatively consistent with LAI estimations using the stratified clipping method for every cultivar and treatment. The parameters calculated using the statistical moment equations numerically showed the changes in LAI vertical distribution with plant growth up to the heading stage. The differences in the parameters also quantified the effect of cultivar, fertilizer, and plant density treatments. These results suggest that the non-destructive stratified measurements and the statistical moments evaluated in this study provide quantitative, reliable information on the dynamics of LAI vertical distribution. The method is expected to be utilized by researchers in various research fields sharing common interests.

## Introduction

Crop canopy structure depends on genotypic characteristics and crop physiological and biochemical processes as well as its planting pattern and growth status^1^. Canopy structure directly influences light distribution and leaf physiological characteristics in crop canopies^2,3^. Leaves and other photosynthetic organs in crop canopies serve as both solar energy collectors and exchangers in the plant community^4^. Evaluation of plant type is important in plant breeding, and analysis of growth dynamics has been recommended for further crop improvement^5^. Specifically, leaf area index (LAI) [m^2^ m^−2^] is one of the most important parameters in climatic, ecological and agronomical studies^6^. Because LAI vertical distribution varies among crop species and cultivars, continuous monitoring is an effective method for analyzing differences within crop canopy photosynthesis and biomass productivity.

Since Monsi and Saeki^7^ first applied the Beer-Lambert law describing random distribution of light to predict light transmission in a plant canopy, several studies have investigated the vertical distribution of the leaves of crops such as rice (*Oryza sativa* L.)^8^, maize (*Zea mays* L.)^9^ and soybean (*Glycine max* (L.) Merr.)^10^. Particularly in rice, cultivars varying in leaf angle have been developed after the Green Revolution. In China, erect panicle rice cultivars have been developed, which have become predominant in the Liaoning province^11^. Monitoring and quantifying the dynamics of LAI vertical distribution are considered a key approach to analyze the difference in rice crop growth dynamics and light energy use^12^. However, huge efforts and time-consuming work involving destructive samplings are commonly necessary to conduct measurements of LAI vertical distribution (stratified clipping method); thus, limiting dynamic measurements.

Non-destructive measurement methods using a plant canopy analyzer can be utilized to overcome these disadvantages. Researchers have reported non-destructive measurements of LAI obtained with a plant canopy analyzer in common bean (*Phaseolus vulgaris* L.), cotton (*Gossypium hirsutum* L.), maize (*Zea mays* L.), rice (*Oryza sativa* L.) and soybean (*Glycine max* (L.) Merr.)^13–17^. In fact, in rice, Stroppiana *et al*.^6^ and Sone *et al*.^17^ reported that even among different cultivars and fertilizer levels a plant canopy analyzer can be used to estimate LAI. Hirooka *et al*.^18^ measured LAI frequently with a plant canopy analyzer and then parameterized the characteristics of LAI dynamics using mathematical functions. Continuous monitoring of LAI vertical distribution is also expected to become even more simplified and is non-destructive, when done by a plant canopy analyzer.

In this study, we used an LAI-2200 plant canopy analyzer (LI-COR Inc., Lincoln, Nebraska), which is a non-destructive and non-labor-intensive piece of equipment, to conduct non-destructive stratified LAI measurements. Following this, statistical moment equations were applied to evaluate LAI vertical distribution. For this purpose, field experiments were conducted with 5 rice cultivars under 2 fertilizer treatments in 2013 and with 3 cultivars under 3 plant density treatments in 2014. In particular, LAI-2200 shows improvement over the earlier model, LAI-2000^19^. In this study, we proposed a novel method to characterize LAI vertical distribution of rice utilizing a plant canopy analyzer, LAI-2200. This method is expected to be utilized by researchers of various fields in which growth dynamic study is important.

## Results

### Validation of the stratified LAI readings of the plant canopy analyzer

**Figure 1:**
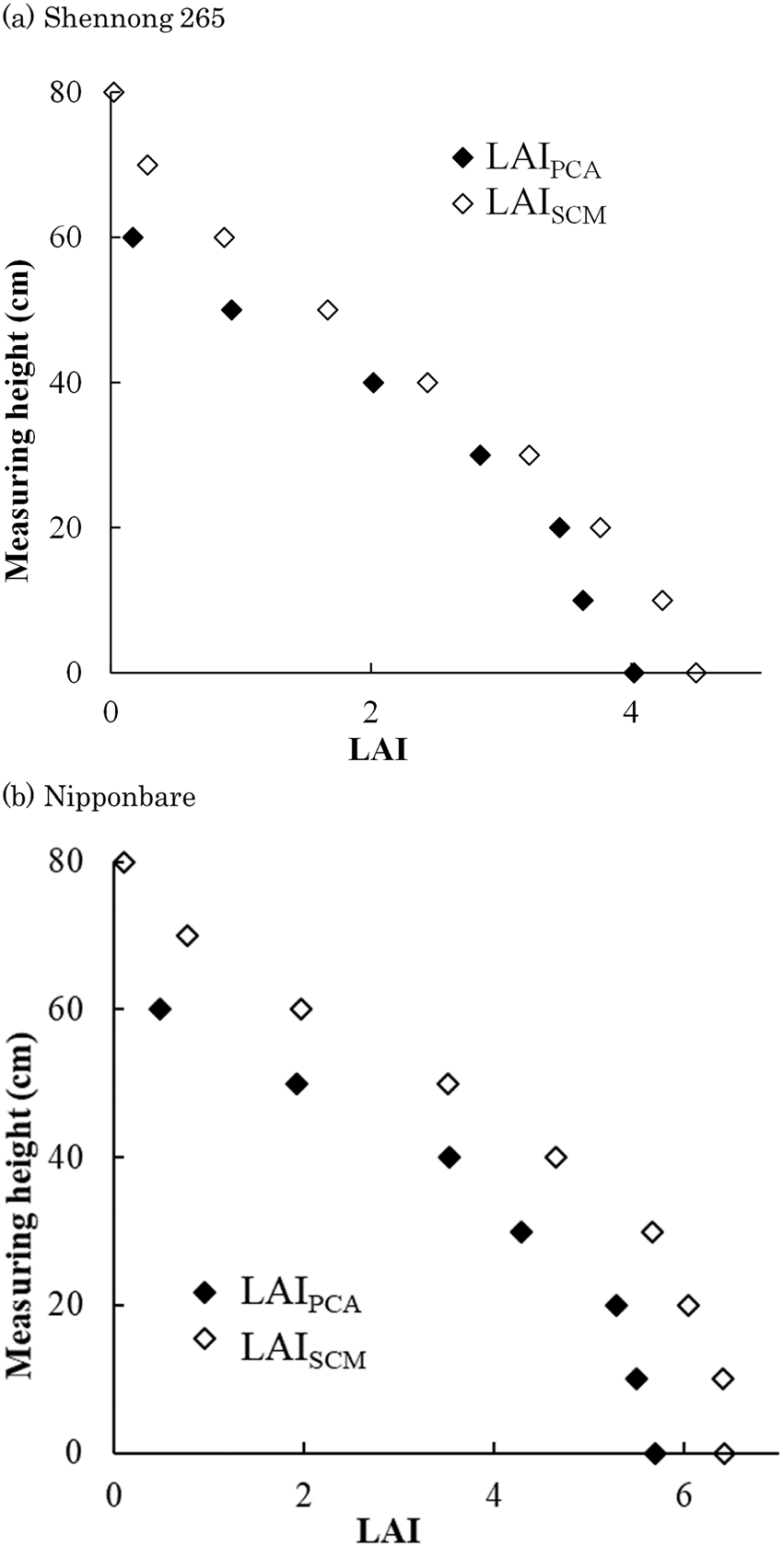
The difference in LAI between LAI_PCA_ and LAI_SCM_ for (**a**) Shennong 265 and (**b**) Nipponbare. LAI_PCA_: LAI readings of a plant canopy analyzer at the measuring height. LAI_SCM_: sum of LAI above the measuring height measured using the stratified clipping method. The measuring height is the height from the ground.

**Figure 2:**
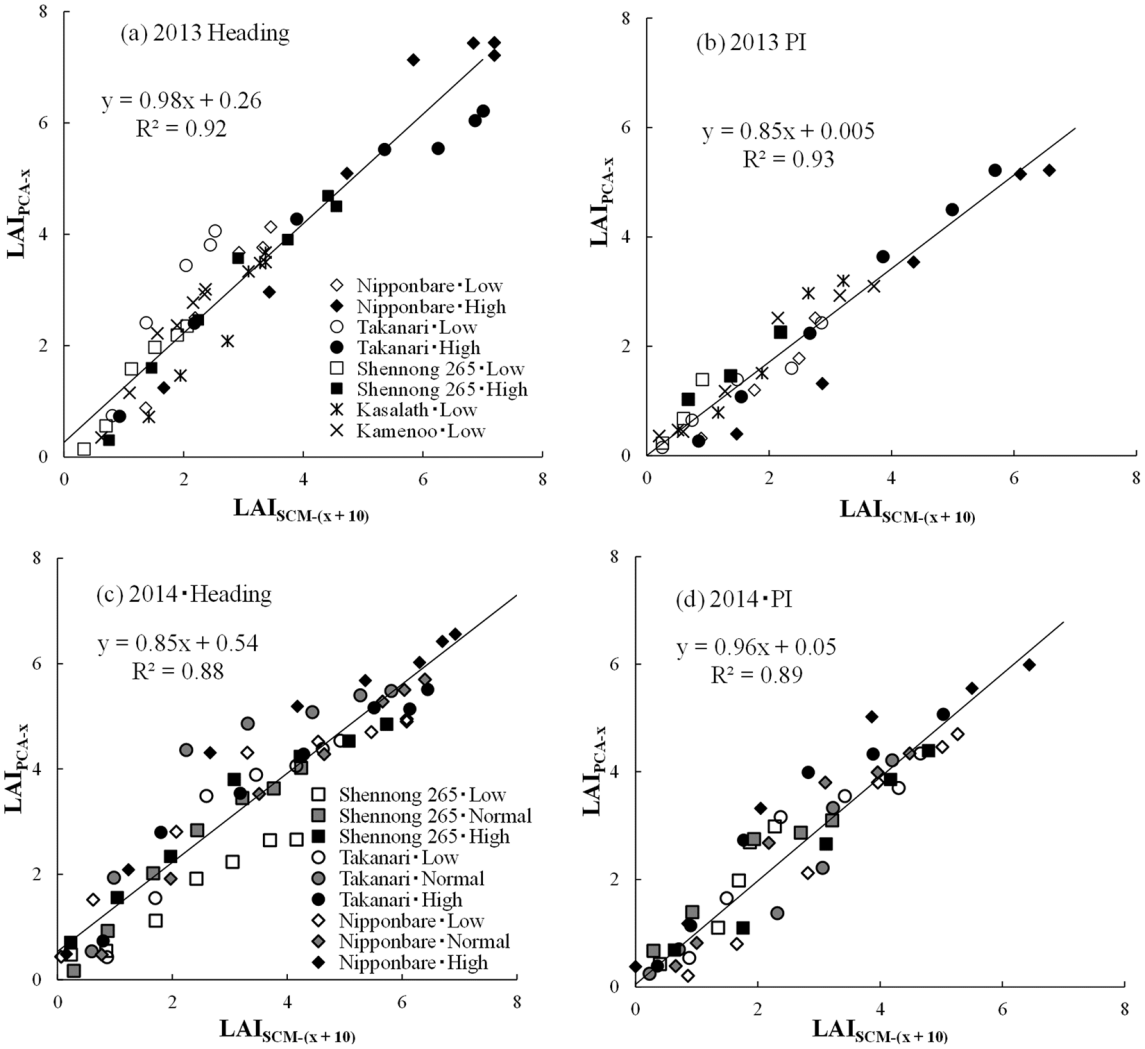
The relationship between the LAI_PCA-x_ and the LAI_SCM-(x + 10)_ (**a**) at heading in 2013, (**b**) at panicle initiation (PI) in 2013, (**c**) at heading in 2014 and (**d**) at PI in 2014. Low and High represent the fertilizer levels in (**a**) and (**b**). Low, Normal and High represent the plant density levels in (**c**) and (**d**). The solid line is the regression line.

Figure 1 shows the difference between LAI readings from a plant canopy analyzer (LAI_PCA_) at each measuring height and the sum of the LAI above the measuring height according to the stratified clipping method (LAI_SCM_). The results show that the LAI_PCA_ measured at x cm height (LAI_PCA-x_) corresponded well with the LAI_SCM_ above the height of (x + 10) cm (LAI_SCM-(x + 10)_). Figure 2 shows the relationship between LAI_PCA-x_ and LAI_SCM-(x + 10)_ at the heading and panicle initiation (PI) stages in 2013 and 2014 ((a) Heading, 2013; (b) PI, 2013; (c) Heading, 2014; and (d) PI, 2014). The dates of PI and heading stage are shown in the supplemental file (Supplemental 1). The stratified LAI_PCA-x_ was relatively consistent with the LAI_SCM-(x + 10)_ at both stages and in both years (R^2^ > 0.85) (Fig. 2). The measurement error differed slightly among cultivars, treatments, and growth stages. The root mean square error (RMSE) and relative root mean square error (rRMSE) between the LAI_PCA-x_ and the LAI_SCM-(x + 10)_ of all plots were 0.614 and 0.216, respectively (Table 1).

**Table 1:** Differences in root mean square error (RMSE) and relative root mean square error (rRMSE) between LAI calculated using a plant canopy analyzer (LAI_PCA-x_) and LAI calculated using the stratified clipping method (LAI_SCM-(x + 10)_) among years, stages, cultivars, fertilizer treatments and plant density treatments.

		RMSE	rRMSE
All		0.614	0.216
Year			
	2013	0.518	0.219
	2014	0.637	0.202
Cultivars			
	Shennong 265	0.473	0.220
	Nipponbare	0.702	0.196
	Takanari	0.694	0.230
	Kasalath^1)^	0.441	0.161
	Kamenoo^1)^	0.515	0.299
Fertilizer			
	High	0.629	0.166
	Low	0.645	0.381
Plant density			
	High	0.656	0.220
	Middle	0.594	0.216
	Low	0.656	0.227
Stages			
	PI^2)^	0.552	0.228
	Heading	0.676	0.206

^1)^Only under low fertilizer levels in 2013.

^2)^PI represents panicle initiation.

### Parameter estimation for leaf area distribution

**Figure 3:**
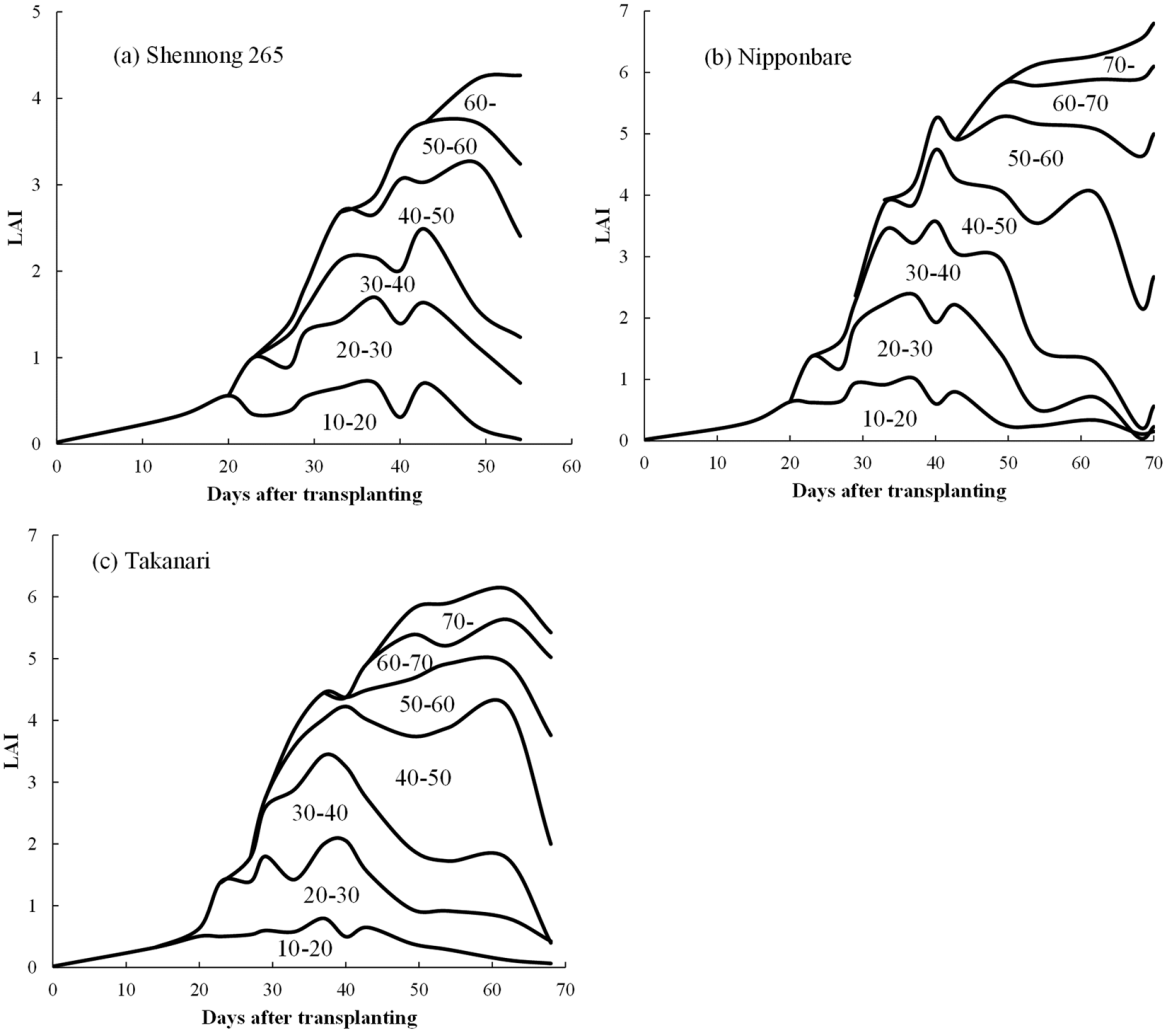
The changes in LAI vertical distribution with plant growth up to heading on the basis of LAI_PCA_ (smooth line of scatter plots generated using Microsoft Excel 2010). (**a**) Shennong 265, (**b**) Nipponbare and (**c**) Takanari under high fertilizer treatment in 2013.

**Figure 4:**
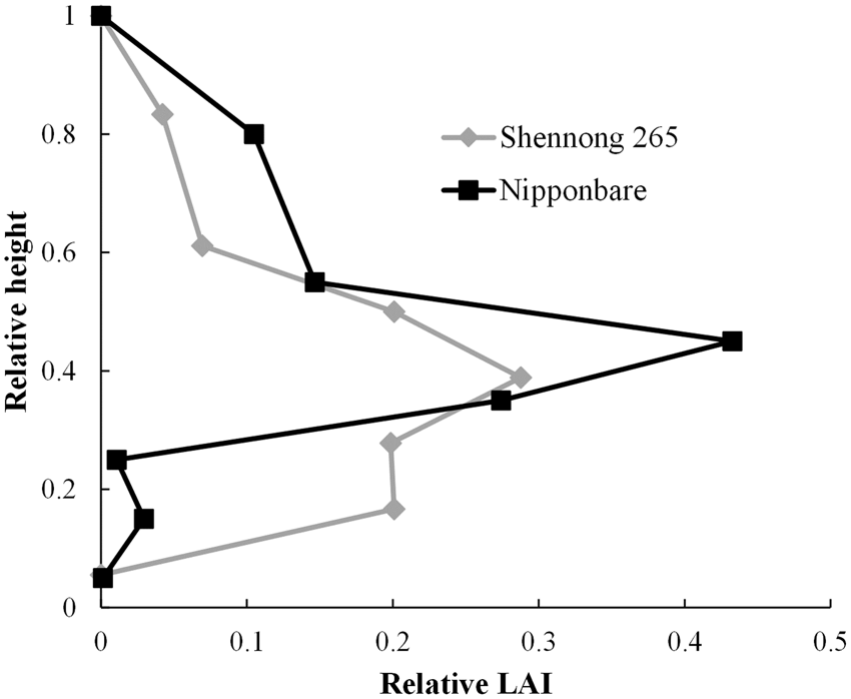
The LAI vertical distribution of Shennong 265 and Nipponbare at heading under high fertilizer treatment in 2013. The height and LAI are shown as relative values: relative height = height of the center of the layer/canopy height; relative LAI = LAI in the layer/total LAI.

Figure 3 shows the results of the periodical change in every 10 cm of stratified LAI_PCA_ with plant growth for cultivars Shennong 265, Nipponbare, and Takanari under high fertilizer (HF) levels in 2013. During the growth period, the LAI vertical distribution of the erect panicle type of rice (Shennong 265) was observed to be relatively uniform, with this tendency becoming remarkable around heading; however, the LAI of the Nipponbare cultivar showed a non-uniform distribution: higher strata had larger LAI (Fig. 4).

In this study, four parameters (a_1_ − a_4_) related to LAI vertical distribution were calculated using the statistical moment equations (Eqs (1–5)) based on the LAI_PCA_. These results are provided in the accompanying supplemental file (Supplemental 2). The first parameter, a_1_, the mean LAI vertical distribution, ranged from 0.295 (Shennong 265 under low fertilizer (LF) levels at 2 weeks before heading (2WBH)) to 0.559 (Takanari under HF levels at heading); second, a_2_, the variance of the LAI vertical distribution, ranged from 0.023 (Nipponbare under HF levels at heading) to 0.100 (Shennong 265 under HF levels at 2WBH). Next, a_3_, the skewness of the LAI vertical distribution, ranged from −0.072 (Shennong 265 under LF levels at 2WBH) to 0.016 (Shennong 265 under normal plant density (ND) levels at 1 week before heading (1WBH)). Finally, a_4_, the kurtosis of the LAI vertical distribution, ranged from 1.55 (Nipponbare under LF levels at 1WBH) to 4.28 (Takanari under ND levels at heading). Parameters a_1_ and a_2_ showed high correlations with a_3_ and a_4_, respectively (Table 2). Further, a_2_ also showed a significant correlation with both a_1_ and a_3_, although the corresponding correlation coefficients were lower (Table 2).

**Table 2:** Correlation coefficient between parameters calculated using statistical moment equations (Eqs (1–5)) (n = 68). a_1_, a_2_, a_3_ and a_4_ represent the mean, variance, skewness and kurtosis of LAI vertical distribution, respectively.

	a_1_	a_2_	a_3_	a_4_
a_1_	1			
a_2_	−0.383**	1		
a_3_	0.789**	−0.386**	1	
a_4_	0.234	−0.833**	0.167	1

^**^At 1% significance level.

The results of ANOVA and the means of the representative parameters are shown in Tables 3 and 4. All parameters, a_1_, a_2_, a_3_ and a_4_, significantly changed with plant growth stage. Both, in 2013 and 2014, a_1_, a_3_ and a_4_ became higher with plant growth, whereas a_2_ became lower. Mean LAI vertical distribution, a_1,_was significantly higher under HF levels than under LF levels and that of Shennong 265 was significantly lower than that of Nipponbare both in 2013 and 2014. The a_2_ of Shennong 265 was significantly higher than that of Nipponbare and Takanari both, in 2013 and 2014. The a_3_ of Shennong 265 was significantly lower than that of Nipponbare and Takanari, in 2013. The a_4_ of Shennong 265 was significantly lower than that of Nipponbare and Takanari, and that under HF levels was significantly higher than under LF levels.

**Table 3:** The results of analysis of variance (ANOVA) of the four parameters of LAI vertical distribution calculated using statistical moment equations in 2013. The values are average, which followed by the same letters indicate no significant difference at 5% significance level.

	a_1_	a_2_	a_3_	a_4_
Stage				
PI	0.351a	0.046a	−0.043	2.61a
2WBH	0.388b	0.050a	−0.028	2.63a
1WBH	0.429b	0.040ab	−0.017	2.57a
Heading	0.441c	0.030b	−0.007	3.53b
Cultivar				
Shennong 265	0.358a	0.050a	−0.039a	2.70
Nipponbare	0.421b	0.036b	−0.016b	2.87
Takanari	0.427b	0.039b	−0.015b	2.94
Fertilizer				
Low	0.378	0.043	−0.033	2.64
High	0.426	0.041	−0.014	3.03
Stage	**	*	**	**
Cultivar	**	*	**	ns
Fertilizer	**	ns	**	*
S × C	**	ns	ns	ns
S × F	ns	ns	ns	ns
C × F	ns	ns	*	ns
S × C × F	ns	ns	ns	ns

*At 5% significance level.

**At 1% significance level.

ns, no significant level.

**Table 4:** The results of analysis of variance (ANOVA) of the four parameters of LAI vertical distribution calculated using statistical moment equations in 2014. The values are average, which followed by the same letters indicate no significant difference at 5% significance level.

	a_1_	a_2_	a_3_	a_4_
Stage				
PI	0.416a	0.036a	−0.018a	3.07
2WBH	0.427ab	0.033ab	−0.012ab	3.13
1WBH	0.445bc	0.033ab	−0.002bc	3.10
Heading	0.499 cd	0.031b	−0.004b	3.34
Cultivar				
Shennong 265	0.431a	0.039a	−0.006	2.74a
Nipponbare	0.450b	0.030b	−0.007	3.35b
Takanari	0.422a	0.031b	−0.012	3.40b
Density				
Low	0.426	0.036a	−0.010	3.05
Normal	0.432	0.032b	−0.010	3.25
High	0.444	0.033ab	−0.005	3.18
Stage	**	*	**	ns
Cultivar	**	**	ns	**
Density	ns	*	ns	ns
S × C	**	*	ns	*
S × D	ns	ns	ns	ns
C × D	ns	ns	ns	ns
S × C × D	ns	ns	ns	ns

*At 5% significance level.

**At 1% significance level.

ns, no significant level.

The a_1_ values showed a significant interaction between growth stage and cultivar both, in 2013 and in 2014. The a_1_ of Shennong 265 was more stable with plant growth than that of Nipponbare and Takanari. Similarly, a_2_ and a_4_ also showed a significant interaction between stage and cultivar, although only in 2014. The a_2_ and a_4_ of Shennong 265 were more variable with plant growth than the corresponding values of Nipponbare and Takanari. Parameter a_3_ showed a significant interaction between cultivar and fertilizer in 2013. Furthermore, a_3_ of Shennong 265 and Takanari was higher under HF levels than under LF levels; whereas a_3_ of Nipponbare under HF levels was almost the same as that under LF levels. These results are shown in the supplemental file (Supplemental 2).

## Discussion

In this study, we performed non-destructive stratified measurements using LAI readings from an LAI-2200 plant canopy analyzer (LI-COR Inc., Lincoln, Nebraska). Figures 1 and 2 show that the LAI_PCA_ closely corresponded to the LAI_SCM_ for every treatment and cultivar, suggesting that the LAI vertical distribution can be evaluated by using the stratified LAI readings of a plant canopy analyzer. The measurement error (rRMSE) between LAI_PCA-x_ and LAI_SCM-(x+10)_ of all plots was 21.6%, which is similar to values obtained in previous measurements in rice canopies^6,13,18^. The measurement error differed slightly among years, growth stages, cultivars, fertilizer levels and plant density levels (Table 1). Although the measurement error could not be ignored, parameterization using statistical moment equations for every 10 cm of stratified measurements of a plant canopy analyzer is supposed to reduce the effect of measurement error. Thus, the consecutive monitoring of LAI vertical distribution followed by parameterization using statistical moment equations are supposed to decrease error variance. In this study, LAI_PCA_ was relatively consistent with LAI_SCM_ when a 10-cm difference was considered. As the viewing angle was 148°, and the thickness of the lens was 3 cm, LAI-2200 could not evaluate just above the measurement point.

Hirooka *et al*.^18^ used a logistic equation to quantify LAI dynamics. In this study, on the other hand, statistical moment equations were used to analyze differences in LAI vertical distribution. The moment equations represent the mean densities and spatial covariance^20^ and may be able to predict spatial characteristics of different cultivars and under different treatments using stratified LAI measurements. The parameters calculated by moment equations in this study did not show significant interaction effects except for the interaction between cultivar and growth stage (only a_3_ showed the interaction effect between cultivar and fertilizer) (Tables 3 and 4). HF treatment in 2013 and ND treatment in 2014 were similar and the pattern of LAI vertical distribution was almost the same. Cultivar Shennong 265 showed uniform distribution, compared to Nipponbare and Takanari both, under HF and ND treatments. These results show that the parameters calculated from the statistical moment equations were more stable for evaluating the cultivar characteristics or the effect of the treatments.

The skewness (a_3_) and kurtosis (a_4_) of the LAI vertical distribution were closely associated with the mean (a_1_) and variance (a_2_) of the LAI vertical distribution, respectively. In this study, the mean and skewness parameters were defined as the center of the LAI vertical distribution and the variance and kurtosis parameters were defined as the uniformity of the LAI vertical distribution. All parameters (a_1_, a_2_, a_3_ and a_4_) varied with each growth stage; thus, they showed numerically the changes in LAI vertical distribution with plant growth up to heading stage. Previous studies also reported that rice canopy structure parameters, such as LAI vertical distribution and extinction coefficient do change with plant growth^21,22^. Both in 2013 and 2014, the center of the LAI vertical distribution became higher with plant growth, whereas the uniformity of LAI vertical distribution became lower. The change in characteristics of LAI vertical distribution with plant growth is considered important for analyzing the processes of dry matter production.

In order to evaluate the genotypic effect, fast and numerical phenotypic analysis is required in terms of bioinformatics^23^. This study focused on the erect panicle type of rice as an example. The erect panicle type of rice generally provides a genetic repository for increasing biomass and harvest index, offering a sustainable yield improvement option for future breeding programs^24^. In this study, cultivar Shennong 265 showed markedly different characteristics of LAI vertical distribution compared with cultivars Nipponbare and Takanari. According to previous studies, erect panicle types of rice have high leaf photosynthetic capacity^25^ and achieve very high yield under high nitrogen conditions^24,26^. Especially, Shennong 265, an erect panicle rice cultivar, showed higher yield potential, such as high nitrogen uptake ability and radiation use efficiency^27^. This might result in the characteristic LAI vertical distribution of an erect panicle rice cultivar. However, little is known about the leaf canopy structure of the erect panicle type of rice despite the importance of canopy structure in determining rice productivity. In Shennong 265, the center of the LAI vertical distribution is lower, whereas the uniformity of LAI vertical distribution is higher. Quantification of these characteristics might help us understand the factors governing the high yield potential, nitrogen uptake ability and radiation use efficiency of erect panicle type of rice cultivar.

Leaf canopy structure is altered by cultivation management^28,29^. Cultivation management, including fertilizer and planting density treatments, also affected LAI vertical distribution in this study. Improvement of cultivation management is required, which will be attained by analyzing the effect of the interaction between cultivar and management^30^. Three parameters (a_1_, a_3_ and a_4_) showed significant differences between fertilizer levels, and one parameter (a_2_) showed a significant difference among plant density levels. The parameter shows that high fertilizer and plant density leads a non-uniform LAI distribution: higher strata had larger LAI. These results strongly agree with previous studies, in which, high nitrogen fertilizer application increased leaf biomass of the uppermost canopy layers^31^, and low plant density leads to uniform LAI vertical distribution^32^. Further studies are necessary to evaluate the effects of other cultivation management techniques, such as planting method (transplanting/direct seeding) and water environment, on LAI vertical distribution.

## Conclusion

In this study, we proposed a novel method to characterize canopy structure of crops by utilizing an LAI-2200 plant canopy analyzer (LI-COR). The non-destructive stratified measurements and parameterization using statistical moment equations revealed that the characteristics of LAI vertical distribution vary with growth stage, cultivar and cultivation management. This method also showed an interaction between rice cultivar and growth stage. These results suggest that the non-destructive stratified measurements and the statistical moments evaluated in this method provide quantitative information on LAI vertical distribution. This evaluation method of LAI vertical distribution is also considered to be applicable to many cultivars under various conditions, as the method facilitates non-destructive stratified measurements in many plots. Therefore, this method is expected to be utilized by researchers in various fields. Information regarding LAI vertical distribution might help us analyze the effect of canopy structure on photosynthetic ability and dry matter productivity.

## Methods

### Study site and experimental design

Field experiments were conducted in paddy fields at the experimental farm of the Graduate School of Agriculture, Kyoto University (35°02′N, 135°47′E, 65 m altitude) in 2013 and 2014.

In 2013, five cultivars were selected for the experiment to cover various characteristics of canopy structure: Shennong 265 is an erect panicle type of japonica rice cultivar^33^; Nipponbare and Kasalath are standard cultivars of japonica and indica rice, respectively^34^; Takanari is a high-yielding indica cultivar; and Kamenoo is a traditional japonica cultivar. Twenty-nine-day-old seedlings were transplanted on 6 June. Each plot was 12.15 m^2^ (4.5 m × 2.7 m), and the planting density was 22.2 plants per m^2^ (0.3 m × 0.15 m); there was one plant per hill. For the low fertilizer treatment (LF), Eco-long (JCAM AGRI), a slow release fertilizer, was applied at rates of 3.00, 2.36, and 2.79 g m^−2^ for N, P_2_O_5_, and K_2_O, respectively. The same fertilizer was applied at rates of 12.00, 9.43, and 11.14 g m^−2^ for N, P_2_O_5_, and K_2_O, respectively, for the high fertilizer-nitrogen treatment (HF). Additionally, 5 g m^−2^ of LP cote (JCAM AGRI), a coated nitrogen fertilizer, was applied to the HF level as a basal fertilizer. The tall cultivars Kamenoo and Kasalath were grown only under LF levels to avoid lodging.

In 2014, three cultivars, Shennong 265, Nippobare and Takanari, were grown, and eco-long was applied at rates of 20.00, 16.67, and 19.00 g m^−2^ for N, P_2_O_5_, and K_2_O, respectively. Three plant density treatments, a high plant density (HD) level (44.4 plants m^−2^), a normal plant density (ND) level (22.2 plants m^−2^) and a low plant density (LD) level (16.7 plants m^−2^), were tested. Twenty-eight-day-old seedlings were transplanted on 5 June. Each plot was 10 m^2^ (2.4 × 4.2 m).

In 2013 and 2014, a randomized block design was established, with 3 replications; water, weeds, insects and disease were controlled as required to prevent yield loss. In order to eliminate the effect of fertilizer treatment in 2013, barley was cultivated in the field between 2013 and 2014.

### Measurements

The LAI_PCA_ was measured one or two times a week beginning two weeks after transplanting through heading, using an LAI-2200 plant canopy analyzer (LI-COR Inc., Lincoln, Nebraska), every 10 cm of vertical height in the canopy at each plot. The measurements were conducted under scattered light conditions, such as after sunrise, before sunset or during overcast days, in single-sensor mode in a sequence of two measurements above followed by four measurements in the canopy of each plot. To reduce the influence of the adjacent plots and of the operator, a 90° view-cap was applied to the optical sensor. Theoretically, the plot size in this study may be enough for LAI-2200 measurements.

Stratified clipping was conducted for two hills at panicle initiation and at heading in one replication, in order to validate the stratified measurements with the plant canopy analyzer. The depth of each stratum was fixed at 10 cm. Plant samples were harvested from an area where it was expected that harvesting would not affect the measurements of the plant canopy analyzer. The samples were chosen to represent the rice canopy based on the number of tillers among 12 plants in the area of measurements of the plant canopy analyzer. The rice plant samples from each stratum were separated into green leaf blades and stems (culms, panicles and dead tissues). Leaf area (LA) was destructively measured for green leaf blades using an area meter (LI3000, LI-COR). The leaf area index (LAI_SCM_) was calculated by dividing the destructively measured LA by the planting area.

### Data analysis

LAI vertical distribution was analyzed for the LAI_PCA_ by calculating 4 parameters in this study. The four parameters (a_1_, a_2_, a_3_, a_4_) describing the LAI vertical distribution were obtained using the following five statistical moment equations:

1 $${a}_{1}=\sum _{i=1}^{k}{h}_{i}\,LA{I}_{i}$$

where a_1_ represents the mean of the LAI vertical distribution, i represents each stratum, h_i_ represents the relative height (0: the bottom; 1: the top of canopy) of center of the i-th stratum and LAI_i_ represents relative LAI (1: total LAI) of the i-th stratum;

2 $${m}_{r}=\sum _{i=1}^{k}{({h}_{i}-{a}_{1})}^{r}LA{I}_{i}$$

where m_r_ represents the r-th moment;

3 $${a}_{2}={m}_{2}$$

where a_2_ represents the variance of LAI vertical distribution;

4 $${a}_{3}={m}_{3}/{({m}_{2})}^{3/2}$$

where a_3_ represents the skewness of LAI vertical distribution; and

5 $${a}_{4}={m}_{4}/{{m}_{2}}^{2}$$

where a_4_ represents the kurtosis of LAI vertical distribution.

The experimental plots were arranged in a randomized complete block design with 3 replications. The main treatments included fertilizer level in 2013 and plant density level in 2014, whereas the sub-treatments included cultivars. Three-way analysis of variance (ANOVA) was applied to the parameters to test the main effects and their interactions (Eq. (6); 2013, Eq. (7); 2014):

6 $${\rm{Parameters}}={\rm{Stage}}\,({\rm{S}})+{\rm{Cultivar}}\,({\rm{C}})+{\rm{Fertilizer}}\,({\rm{F}})+{\rm{S}}\times {\rm{C}}+{\rm{C}}\times {\rm{F}}+{\rm{F}}\times {\rm{S}}+{\rm{S}}\times {\rm{C}}\times {\rm{F}}$$

7 $${\rm{Parameters}}={\rm{S}}+{\rm{C}}+{\rm{Density}}\,({\rm{D}})+{\rm{S}}\times {\rm{C}}+{\rm{C}}\times {\rm{D}}+{\rm{D}}\times {\rm{S}}+{\rm{S}}\times {\rm{C}}\times {\rm{D}}$$

## Electronic supplementary material

Supplement 1, Supplement 2
